# On Retention of Urine and Its Treatment

**Published:** 1898-10-08

**Authors:** J. Lacy Firth

**Affiliations:** Assistant Surgeon to the Bristol General Hospital.


### Medical Progress and Hospital Clinics.

### ON RETENTION OF URINE AND ITS TREATMENT.

By J. Lacy Firth, M.D., M.S., F.R.C.S., Assistant Surgeon to the Bristol General Hospital.

In all cases of retention of urine, with the exception of some in which a stone is impacted in the urethra of boys, the proper treatment, when possible, is to pass a catheter and to draw off the urine. In many cases nothing is easier than to relieve the trouble in this way. In other cases, however, it is difficult to pass a catheter, and certain methods have to be used to surmount the difficulties. A catheter can be passed easily in those cases of retention due to shock, fevers such as typhoid, acute and chronic (*e.g.*, tabes and myelitis) diseases of the central nervous system, peritonitis, and to certain operations, *e.g.*, those on the anus, and those for the cure of hernia, hydrocele, and varicocele. In this class of case select a highly polished Jacques catheter with bevelled eye. If there is any difficulty with that use an English silk-web catheter, with solid end, or French gum-elastic olivary catheter. Whichever of these instruments is used, and there is little to choose between them, let it be of medium size, 7-9 English, and well lubricated with oil.

The cases in which difficulty is often met with in passing a catheter are those due to urethral obstruction, or to pressure on the neck of the bladder. Urethral obstruction may arise from prostatitis, stricture, senile enlargement of the prostate, and from impacted stone in or rupture of that channel. Pressure on the bladder neck may be due to uterine displacements, especially retroversion and flexion of the gravid uterus, rectal collections, pelvic growths, and pelvic hydatids.

In every case of retention of urine it is imperative to relieve the condition early, for in addition to the physical and mental distress which is usually present, there is great danger of the urinary organs being permanently injured, causing much future misery or even speedy death. These dangers are, partial or complete atony of the bladder, rupture of the bladder, or of the urethra with intra or extra-peritoneal extravasation of urine, and damage to the kidney from back pressure. Also that most distressing malady, tuberculosis of the bladder, seems in some cases to have been started by retention of urine. Sir H. Thompson states that he has seen patients who have been unable to empty the bladder for years after prolonged retention. Rupture of the bladder from retention is rare, but many cases are on record. I may mention one recorded of a man of sixty. There was retention from stricture, and the bladder reached the level of the umbilicus. No instrument could be passed, so ether was given. During the restlessness which the ether produced, the bladder tumour disappeared. The man died in four hours with five pints of urine in the peritoneum. In some of the cases, straining at stool has been the immediate cause of the rupture of the distended bladder. Rupture of the urethra behind a stricture, either with or without previous ulceration there, is much commoner than rupture of the bladder, and is often fatal from the cellulitis which follows the urinary extravasation. The great importance of securing early relief in retention of urine is thus very obvious.

We will now consider the treatment of retention in more or less difficult cases. In the first place, it may be stated, that whenever a distended bladder can be felt above the pubes, it can be emptied with an aspirator, either Potain's or Dieulafoy's, preferably the former, with very little risk. The knowledge of this fact will give a confidence to the practitioner undertaking the treatment of such cases which will be of much benefit both to him and to his patients. Of course, the bladder should be aspirated only if a catheter cannot be passed.

Taking the cases of retention as met with at different age periods, we have first those in boys; they are usually due to the impaction of a stone in the urethra, but may be due to a spasmodic condition. In the latter case a hot bath or hot enema will often be sufficient to procure relief, and if not there will be no difficulty in passing a small soft catheter. If a calculus is present it is likely to be near the meatus or in the scrotal part of the urethra, these being narrow parts of the passage. As the urethra is very delicate and sensitive an anæsthetic should be given. Then if the stone is near the meatus, incise that orifice downwards, and, after injecting some warm oil with a syringe, make gentle endeavours to extract the stone with a Lister's sinus forceps, or some other narrow-bladed forceps, until successful. If the stone is in the penile urethra, but in front of the scrotum, a clean incision should be made on to the stone from the under surface, and the wound closed afterwards by sutures. Should the stone be in the scrotal part of the urethra, or behind the scrotum, it will be best to inject oil, and then, with a No. 5 or 6 gum elastic or silver catheter, gently tap the stone until it recedes into the bladder, where it can be crushed with a lithotrite and evacuated. If the lithotrity has to be postponed, the patient must pass urine lying on his back, to avoid re-impaction.

Retention from rupture of the urethra may, of course, be met with at any age. The rupture is generally incomplete, involving the floor and lateral walls. Unless there is fracture of the pelvis the laceration is usually just in front of the triangular ligament, but when there is such a fracture the membranous portion often suffers. A silver catheter should be used, and attempts made to pass it into the bladder by keeping it in contact with the roof of the urethra, thus avoiding the lacerations. If passed successfully, the catheter must be tied in, and, further, in the majority of cases it is advisable to make a perineal incision to insure efficient drainage of the bruised tissues. Otherwise, urine will escape by the side of the catheter, and may lead to extensive suppuration. If a catheter cannot be successfully passed in this way, you must cut down from the perineum upon the end of a grooved staff passed as far as the seat of obstruction, the patient being in the lithotomy position. The two ends of the urethra where it has been divided will be found through the incision, and a catheter guided into the bladder. The advisability of then suturing the wound in the urethra is an open question.

In the young adult, retention is most frequently the result of prostatic inflammation following a recent attack of gonorrhœa. The history and the presence of urethral discharge will easily determine the diagnosis. A soft instrument of size No. 6 E, either a Jacques or a silk-web, should be selected, for there is no true stricture, the retention being due to the swollen mucous membrane of the prostatic urethra and prostatic tissue proper. After drawing off the urine clear out the rectum and give a hot enema and a morphia suppository.

Stricture of the urethra, as a cause of retention, is usually met with in patients above thirty years of age, and there is a history of urinary difficulties having existed for some time previously. These patients with stricture do not suffer the same degree of agony from retention as the gonorrhœal cases just considered. They are accustomed to difficulties in urination, and their bladders are less sensitive. Select a small French gum-elastic catheter, a No. 3 or 4, and after injecting a syringeful of warm oil, endeavour to pass it in the usual way. If this fails a silver catheter must be tried. But a small silver catheter is obviously an instrument well calculated to injure the delicate urethra, and it is better, therefore, to be provided with one of No. 6 size, which screws into the end of a Teevan's guide. The latter is passed first, then the catheter screwed on, and made to follow in the same track. If you fail with these instruments, aspirate the bladder above the pubes. Whenever catheterisation has been difficult, though successful, the instrument should be tied in. It will then dilate the stricture, and will save one from the possible mortification of failing to pass an instrument if called upon to do so for a recurrence of the retention.

Lastly, we come to those cases of retention due to senile enlargement of the prostate. The patients are over 50, and in uncomplicated cases there will be no history of urinary troubles before that age. The retention may be caused by a valve-like action of that portion of the prostate which projects into the bladder. It may therefore be at the very neck of the bladder that the obstruction in passing the catheter is found. More often, however, the retention is due to sudden congestion of the enlarged prostate induced by exposure to cold or by dietary excess. The prostate, as felt per rectum, may be hardly appreciably enlarged, or may be immensely enlarged, the finger being unable to reach above it. To relieve the retention, try first a Jacques catheter. If that cannot be passed, try a silk-web Coudée catheter with a blunt elbow or a sharply curved one. If these fail try a bi-Coudée. When the enlargement felt per rectum is considerable, an English gum-elastic instrument, with a good quarter-circle curve, used with a stylet, can often be successfully passed. Sometimes such a catheter will more easily ride over the obstruction at the bladder neck when it has been kept over-curved on a stylet for a month beforehand, and if it be passed without a stylet. Thus prepared, the catheter has a tendency to resume its curve when it meets with an obstruction in the urethra, and so gains the bladder. If the above instruments fail to pass, a silver catheter must be tried. These are made with various curves, and are two to four inches longer than an ordinary catheter. It is very seldom that the very long instrument of this class is required—the one of which the curve is also a long arc of a large circle—but it may be if the enlargement of the gland is enormous. It is to be remembered, however, that in the treatment of prostatic retention failure has sometimes resulted solely from using an instrument too short to reach into the bladder. All the catheters used for these cases must be large, a No. 10 E being a good size.

If you have great difficulty in passing an instrument, but are finally successful, it will usually be wise to tie it in. But an enlarged prostate, unlike a stricture, is rather damaged than benefited by this treatment, and the rigid instruments inflict the greatest damage.

If no instrument can be passed through the urethra the bladder must be aspirated above the pubes. This procedure is a very valuable one for all those cases of retention of urine in which catheterisation fails after patient and careful trial, and may then be resorted to without fear. If you have to deal with a case in which the urethra is bleeding freely from the previous attempts of others to pass a catheter, you will do well to aspirate at once, and so avoid doing further injury to the urethra.

It is true that aspiration is occasionally followed by extravasation of urine along the track of the needle, and by cellulitis and suppuration in front of the bladder. But these events are rare, and may be almost certainly avoided, if due care is exercised in performing the operation. Of course, the skin must be thoroughly cleansed, after shaving. The needle must be rendered aseptic to start with, and if the urine is foul it must be disinfected again before withdrawal. For this purpose inject into the empty bladder a warm solution of perchloride of mercury, 1 in 10,000, through the cannula with a syringe, aspirate again, and repeat this washing out several times, and each time move the end of the tube gently about in the injected fluid. You will thus cleanse both the instrument and the bladder. Also, when withdrawing the cannula, maintain suction in the aspirator so that no urine will escape from its lumen into the tissues. I have seen the bladder aspirated nine times in a week without the least trouble of any kind, and a case is recorded in which the bladder was emptied in this way every day for five weeks, in a man of ninety, with only beneficial results.

With regard to the treatment of retention by the hot bath and opium, it may be said that the method usually involves too much delay. In the case of senile prostate any delay is prejudicial from the atony it induces, and the treatment under consideration is not likely to achieve much. In the gonorrhœal and stricture cases it may relieve the condition, and should certainly be tried, if instruments are not at hand and until they can be obtained.

One other method of treating obstinate retention may be mentioned, viz., that by Cock's operation, in which the urethra, often distended, is opened behind the stricture from the perineum. This somewhat difficult operation will almost never be required if the methods of treatment given above are faithfully carried out.

